# Spatial separation of the cyanogenic β-glucosidase ZfBGD2 and cyanogenic glucosides in the haemolymph of *Zygaena* larvae facilitates cyanide release

**DOI:** 10.1098/rsos.170262

**Published:** 2017-06-28

**Authors:** Stefan Pentzold, Mikael Kryger Jensen, Annemarie Matthes, Carl Erik Olsen, Bent Larsen Petersen, Henrik Clausen, Birger Lindberg Møller, Søren Bak, Mika Zagrobelny

**Affiliations:** 1 Department of Plant and Environmental Sciences and Copenhagen Plant Science Centre, University of Copenhagen, Thorvaldsensvej 40, 1871 Frederiksberg C, Denmark; 2 Department of Bioorganic Chemistry, Max Planck Institute for Chemical Ecology, Hans-Knöll-Straße 8, 07745 Jena, Germany; 3 Department of Cellular and Molecular Medicine, University of Copenhagen, Blegdamsvej 3B, 2200 Copenhagen N, Denmark

**Keywords:** cyanogenesis, β-glucosidase, cyanogenic glucoside, haemolymph, caterpillar

## Abstract

Low molecular weight compounds are typically used by insects and plants for defence against predators. They are often stored as inactive β-glucosides and kept separate from activating β-glucosidases. When the two components are mixed, the β-glucosides are hydrolysed releasing toxic aglucones. Cyanogenic plants contain cyanogenic glucosides and release hydrogen cyanide due to such a well-characterized two-component system. Some arthropods are also cyanogenic, but comparatively little is known about their system. Here, we identify a specific β-glucosidase (*ZfBGD2)* involved in cyanogenesis from larvae of *Zygaena filipendulae* (Lepidoptera, Zygaenidae), and analyse the spatial organization of cyanide release in this specialized insect. High levels of *ZfBGD2* mRNA and protein were found in haemocytes by transcriptomic and proteomic profiling. Heterologous expression in insect cells showed that ZfBGD2 hydrolyses linamarin and lotaustralin, the two cyanogenic glucosides present in *Z. filipendulae*. Linamarin and lotaustralin as well as cyanide release were found exclusively in the haemoplasma. Phylogenetic analyses revealed that *ZfBGD2* clusters with other insect β-glucosidases, and correspondingly, the ability to hydrolyse cyanogenic glucosides catalysed by a specific β-glucosidase evolved convergently in insects and plants. The spatial separation of the β-glucosidase ZfBGD2 and its cyanogenic substrates within the haemolymph provides the basis for cyanide release in *Z. filipendulae*. This spatial separation is similar to the compartmentalization of the two components found in cyanogenic plant species, and illustrates one similarity in cyanide-based defence in these two kingdoms of life.

## 1. Introduction

β-Glucosidases are ubiquitous glycosidases found in all kingdoms of life, involved in various processes such as biomass conversion in microbes, glycoside metabolism, cell wall lignification, phytohormone activation and chemical defence in plants and insects [1–3]. β-Glucosidases are categorized into the glycoside hydrolase families GH1, GH2, GH3, GH5, GH9, GH30 and GH116 [4], with GH1 constituting the largest family, and encompassing most characterized plant β-glucosidases [1]. Many plant species defend themselves against herbivores and pathogens via β-glucosidase-mediated release of toxic aglucones from glucoside precursors, e.g. benzoxazinoid, cyanogenic, iridoid and phenolic glucosides as well as glucosinolates [3]. A similar situation is found in some insect species that sequester or biosynthesize some of the same defence compounds along with the corresponding β-glucosidase [5–11].

Chemical defence mediated by β-glucosidases is an important driver of the herbivore–plant arms race, and is excellently illustrated by the phenomenon of cyanogenesis [12–16], which is the release of hydrogen cyanide (HCN) from cyanogenic glucosides (CNglcs) catalysed by β-glucosidase activity [17]. After cleavage of the glucosyl moiety from CNglcs, the corresponding α-hydroxynitrile dissociates spontaneously at pH values above 6 or via the action of an α-hydroxynitrile lyase into HCN and a keto compound (electronic supplementary material, figure S1). HCN is an acute toxin for eukaryotic organisms due to inhibition of the cytochrome *c* oxidase, the terminal enzyme in the mitochondrial respiratory pathway [18]. In plants, spatial compartmentalization of the two components, i.e. CNglcs and β-glucosidase, on the tissue or cellular level ensures that HCN is only released after tissue disruption due to e.g. herbivore attack [3,9]. Arthropods may also rely on HCN for defence [17,19], and here cyanogenesis may either proceed in special tissues morphologically separated from the rest of the body, such as defence secretions [20–22], or in tissues largely connected to the rest of the body, such as gut and haemolymph. In this case, a prerequisite for cyanogenesis is the immediate enzymatic detoxification of HCN by β-cyanoalanine synthase [23–25]. This ability probably enabled some insects to exploit HCN for their own benefit, as reported from the burnet moth *Zygaena filipendulae* (Lepidoptera, Zygaenidae) that release HCN for defence, development and mating communication [26,27]. The CNglcs in *Z. filipendulae*, linamarin and lotaustralin, are derived from biosynthesis or sequestration, depending on the content in the food plant *Lotus corniculatus* which is polymorphic with respect to levels of CNglcs [7,8,28]. Furthermore, significant amounts of HCN in Z*. filipendulae* are released from crude larval haemolymph, whereas other tissues such as cuticular cavities containing defence droplets do not release HCN *per se* [29]. This scenario renders a haemolymph-based β-glucosidase with activity against CNglcs likely, and accordingly, *Zygaena trifolii* larvae have been shown to harbour cyanogenic β-glucosidase activity in the haemolymph [5]. It is still unknown which gene encodes the putative cyanogenic β-glucosidase and if the gene evolved in convergence or divergence to cyanogenic plant β-glucosidases. Furthermore, the localization of the β-glucosidase and its substrates would unravel whether compartmentalization of the two components occurs in *Zygaena* insects.

Here we identify and characterize a cyanogenic β-glucosidase from larvae of the specialist insect *Z. filipendulae* and elucidate the enzyme's spatial occurrence in comparison to its substrates, showing that cyanogenesis proceeds in the haemoplasma.

## 2. Material and methods

### 2.1. Biological material, haemolymph fractionation and viability stains

Larvae of *Z. filipendulae* and its *L. corniculatus* food plants were collected from a natural population outside Copenhagen, Denmark (55.65° N, 12.30° E). Larvae were kept in plastic boxes and supplied with *L. corniculatus* ad libitum. In order to localize the β-glucosidase, crude haemolymph was collected from punctured prolegs of ice-chilled larvae, and separated into haemoplasma (supernatant) and haemocytes (pellet) by centrifugation for 10 min at 3000*g* and 4°C. The haemocytes were re-suspended in 60 mM citrate buffer pH 6. Viability of the haemocytes was analysed by staining with 2 mM Fluorescein Diacetate (FDA) and monitoring in a fluorescence microscope (Leica DMR). Defence droplets were collected by stroking the larva with a pipette tip, and used immediately in assays before the secretion hardened [29]. All other tissues such as head, gut, integument, Malpighian tubules and fat body were obtained by dissection of ice-chilled penultimate instar larvae followed by washing in 0.9% NaCl to exclude haemolymph contamination. RNA was extracted using the RNAqueous®-Micro Kit (Ambion); cDNA was synthesized using SuperScript™ III Reverse Transcriptase (Invitrogen™).

### 2.2. Identification of β-glucosidase and glucocerebrosidase genes

Candidate genes from the β-glucosidase (GH1) and glucocerebrosidase (GH30) gene families were identified in three different *Z. filipendulae* transcriptome datasets [30,31] by BLAST searches (using BLASTx and BLASTn with default algorithm parameter settings) using selected insect protein sequences from GH1 and GH30 as queries (accession numbers displayed in figure 4). Gene candidates from *Z. filipendulae* were then BLAST searched against the *Z. filipendulae* transcriptomes to ensure exhaustive searches. Approximately 23 β-glucosidases (GH1) and 6 glucocerebrosidases (GH30) were found in all the *Z. filipendulae* transcriptomes. The exact number remains to be determined, since many of the sequences were partial and may belong to the same transcript. Only four of the sequences (*ZfGBA1*, *ZfBGD1*, *ZfBGD2*, *ZfBGD3*) were full length in the 454 transcriptome [30], and thus deemed highly expressed, and used for heterologous expression. Combining all three transcriptomes, it was possible to manually assemble 11 β-glucosidases and 3 glucocerebrosidases to full length, which were subsequently used for phylogenetic analyses.

### 2.3. Molecular cloning and heterologous expression

Since *ZfBGD1* is already characterized [29], the remaining three candidate genes *ZfGBA1*, *ZfBGD2* and *ZfBGD3* were selected for heterologous expression in *Sf*9 cells*.* PCR amplification of the open reading frames were carried out as in [29]. For primers used, see electronic supplementary material, table S1. The products were Sanger-sequenced (European Nucleotide Archive accession number: LT635663 for *ZfBGD2*; LT635664 for *ZfBGD3*; LT635665 for *ZfGBA1*), cloned into the XmaI- and NotI-restriction sites of an insect cell expression vector (pAcGP67A, BD Pharmingen), and subsequently mixed with Baculo-Gold DNA™ (BD Pharmingen) to transfect *Sf*9 insect cells (Life Technologies) for cultivation in Grace media (Gibco®, Life Technologies). Human UDP-N-Acetyl-α-D-Galactosamine:Polypeptide-N-Acetylgalactosaminyltransferase 2 (GalNAc-T2) was expressed as control [32]. The candidate genes *ZfBGD2* and *ZfGBA1* were expressed with and without (*) a predicted native N-terminal targeting signal peptide. *Sf*9 cells were harvested by centrifugation for 2 min at 3000*g* and 4°C and re-suspended in 60 mM citrate buffer pH 6. Viability of the cells after centrifugation was confirmed by staining with FDA and monitoring intact cells in a fluorescence microscope (electronic supplementary material, figure S2). Expression of β-glucosidases was analysed by SDS-PAGE and western blot (electronic supplementary material, file S1 and figure S3). For further details of expression see [33].

### 2.4. Assays for generic and specific β-glucosidase activity, and hydrogen cyanide release

To measure general β-glucosidase activity, re-suspended *Sf9* cells were assayed with the generic substrate 4-methylumbelliferyl β-D-glucopyranoside (MUglc, Sigma M3633) [29]. Cyanogenic activity was assayed using the physiologically relevant substrates linamarin and lotaustralin (both aliphatic) as well as the non-physiologically relevant aromatic CNglc prunasin. All three CNglcs were chemically synthesized [34]. Recombinant ZfGBA1, ZfBGD2 or ZfBGD3 were each incubated with 500 µM of each substrate (up to 10 times higher substrate concentrations were also tested) in 200 µl 60 mM citric acid buffer (pH 6) for 60 min at 37°C. Similarly, crude haemolymph (0.1 µl), boiled haemolymph (5 min 100°C, 10 µl, to de-activate β-glucosidases), haemoplasma (0.1 µl), re-suspended haemocytes (1 µl), and defence droplets (1 µl) were spiked with expressed β-glucosidases and analysed using the same incubation procedure. Hydrolysis products, i.e. aglucones such as 4-methylumbelliferone (MU) or HCN were measured on a microplate reader (SpectraMax M5, Molecular Devices) and quantified based on a corresponding standard curve. For details see ‘β-Glucosidase assays' in [29]. Three types of haemolymph control samples were prepared: 1 ml crude haemolymph (from 16 L7 larvae) was passed through a PD-10 desalting column containing Sephadex™ G-25 medium (GE Healthcare) to prepare (i) haemolymph without CNglcs [29]. To obtain (ii) haemolymph without β-glucosidases, 200 µl crude haemolymph was filtered through a Vivaspin™ 6 sample concentrator (GE Healthcare) with a cut-off at 30 kDa [29]. Haemolymph devoid of both CNglcs and β-glucosidase (iii) was prepared by passing 700 µl of crude haemolymph already filtered through a PD-10 column, through a Vivaspin™ concentrator. All columns were calibrated in 60 mM citrate buffer pH 6 prior to use; column fractions were tested for presence of CNglcs and β-glucosidase activity as described above. Fractions were then tested with approximately 1 µg expressed β-glucosidases, crude haemolymph, boiled haemolymph and defence droplets.

### 2.5. Quantitative real-time PCR

Quantitative real-time PCR (qRT-PCR) was carried out to analyse the mRNA levels of the three β-glucosidase candidate genes (*ZfGBA1, ZfBGD2, ZfBGD3*) in different tissues. Data was quantified relative to the mRNA levels of the reference gene RNA polymerase II 140 kDa subunit (*RpII140-RA*, GenBank accession number KJ192329) [28] using the 2^−ΔΔCt^-method [35]. Reactions were run on a CFX-96 Touch™ Real-Time PCR Detection System (Biorad) using Brilliant III Ultra-Fast SYBR® Green QPCR Master Mix (Agilent Technologies) and cDNA as template. Two technical replicates were analysed from five (crude haemolymph, haemocytes, haemoplasma) or three biological replicates (head, gut, integument, Malpighian tubules, fat body). Technical replicates with a Ct difference of more than 0.5 were repeated. Distilled water as template served as negative control. For primers used, see electronic supplementary material, table S1.

### 2.6. Proteomics

To identify proteins in haemocytes and haemoplasma, protein extracts from both fractions from three different larvae were tryptically digested and desalted. Peptides were separated on a UPLCM system from Waters with a 120 min gradient followed by analysis in a Q-Exactive™ HF Hybrid Quadrupole-Orbitrap mass spectrometer. The gradient used buffer A, 0.1% formic acid in H_2_O, and Buffer B, 0.1% formic acid in ACN (Biosolve UPLC quality). Buffer A is given: 97% 0–14 min (loop online); 97% 14–15 min (loop offline); 97%–70% 15–75 min; 70%–60% 75–90 min; 60%–10% 90–94 min; 10% 94–104 min; 10%–97% 104–105 min; 97% 105–120 min; The HPLC flow rate was 400 nl min^−1^, and the column temperature 50°C. Samples were run on a 200 mm (length) × 75 µm (ID) reversed phase CSH (charged surface hybrid) column (Waters) with a particle size of 1.7 µm. The spectra were recorded in positive ionization mode by applying a voltage of 2.4 kV to the emitter, and measured in the mass range *m/z* 300–1650, using a resolution of 120 000 with an ion time of 100 ms, and a target value of 1E6. Top 15 MS/MS spectra were acquired with a resolution of 15 000 using an ion time of 100 ms and a target value of 1E5 ions. The peptides with a detected charge of 2+, 3+, 4+, 5+, 6+, 7+ or 8+ were selected for the MS/MS acquisition with a width of *m/z* 1.4, and a normalized collision energy (NCE) of 27. Fragmented peptides were dynamically excluded from further MS/MS analysis for 30 s. The search was carried out in Mascot Daemon version 2.5.1 using a *Z. filipendulae* transcriptomic database with 29.395 unique entries created in house. The search settings were as follows: enzyme Trypsin with 1 missed cleavage allowed, Peptide tolerance ±10 ppm and charge 2+ and 3+. MS/MS tolerance was set to 0.5 Da. Carbamidomethyl was set as fixed modification, and oxidation as variable modification. Peptides were accepted as identified with a Peptide Score of 30, and proteins as identified with at least 2 peptides and a false discovery rate of 0.1%. For a rough semi-quantitative evaluation, we used the Mascot protein Score. We verified the sequence coverage and the reliability of the Score manually for the proteins of interest.

### 2.7. Liquid chromatography–mass spectrometry

Liquid chromatography–mass spectrometry (LC-MS) analysis was carried out using re-suspended haemocytes (1 µl), haemoplasma (0.1 µl) and crude haemolymph (0.1 µl). Samples were added to ice-cold 55% methanol (containing 0.1% formic acid and 0.044 mM amygdalin as internal standard) resulting in haemocyte disruption. Analytical LC-MS was carried out as described in [8]. Mass spectral data were analysed with the native data analysis software. Sodium adducts of linamarin (retention time, RT, 2.6 min, [M + Na]^+^at *m/z* 270), lotaustralin (RT 5.5 min, [M + Na]^+^ at *m/z* 284), and amygdalin (RT 6.6 min, [M + Na]^+^ at *m/z* 480) were detected and their RTs compared to authentic standards [34]. Quantification of each compound was based on extracted ion chromatogram (EIC) peak areas compared to calibration curves of linamarin, lotaustralin and amygdalin.

### 2.8. Phylogenetic analysis of β–glucosidases and glucocerebrosidases

Candidate genes were aligned in MEGA7 [36] using MUSCLE [37,38] with default settings, and refined manually (electronic supplementary material, file S2). Representative gene sequences from the moths *Amyelois transitella*, *Bombyx mori*, and *Plutella xylostella*, as well as the butterflies *Danaus plexippus*, and *Papilio polytes*, were downloaded from GenBank following BLAST searches with *ZfGBA1*, *ZfBGD2* and *ZfBGD3* as query sequences (using BLASTx and BLASTn with default algorithm parameter settings). The honeybee *Apis mellifera* was used as outgroup in the glucocerebrosidase phylogenetic tree and *LjBGD2* from *Lotus japonicus* and linamarase from clover *Trifolium repens* [39] in the β-glucosidase tree. Additionally, recently characterized β-glucosidases from the chrysomelid beetles *Chrysomela lapponica*, *Phaedon cochleariae* and *Phyllotreta striolata* were added to the β-glucosidase tree, and the only characterized glucocerebrosidase from insects, DmGba1b from *Drosophila melanogaster*, was added to the glucocerebrosidase tree. Phylogenetic trees were generated in MEGA7 using protein sequence alignments, and the maximum-likelihood method with a JTT model, and a discrete Gamma distribution. This model was chosen based on the model test from the MEGA7 program, where the model with the lowest BIC score (Bayesian information criterion) is considered to describe the observed substitution pattern best. To examine whether candidate genes had been under positive (*ω* values > 1) or negative selection (*ω* values < 1), nonsynonymous to synonymous substitution rate ratios (*ω* = (d*N*/d*S*)) were calculated for codon-based nucleotide alignments with the program *codeml* from the PAML package, v. 4.1 [40,41]. Different maximum-likelihood models of codon substitution were tested to account for variable selection pressures among different amino acid sites. All site models (NSsites) were tested with the settings model = 0. Branch models were also tested (model = 1:b). The remaining settings were default. The different models with different classes of *ω* were compared with likelihood ratio tests [42] to detect if specific regions of the genes had been under positive selection [43].

### 2.9. Investigation of proposed positively selected residues

The ZfBGD2 protein sequence was BLAST searched against the protein data bank (PDB) to identify templates suitable for homology modelling. The two best hits, a β-glycosidase from *Spodoptera frugiperda* (5CG0_A, E-value = 0.0) and a β-glucosidase from *Neotermes koshunensis* (3AHZ_A, E-value = 9.40268 × 10^−127^), were selected based on sequence identity, resolution of the structure and quality of the structural determination. The sequences were aligned pairwise using the MUSCLE algorithm. The alignment and template structure was used by MODELLER [44] to create the homology model.

## 3. Results

### 3.1. ZfBGD2 hydrolyses linamarin and lotaustralin resulting in cyanide release

**Figure 1.**
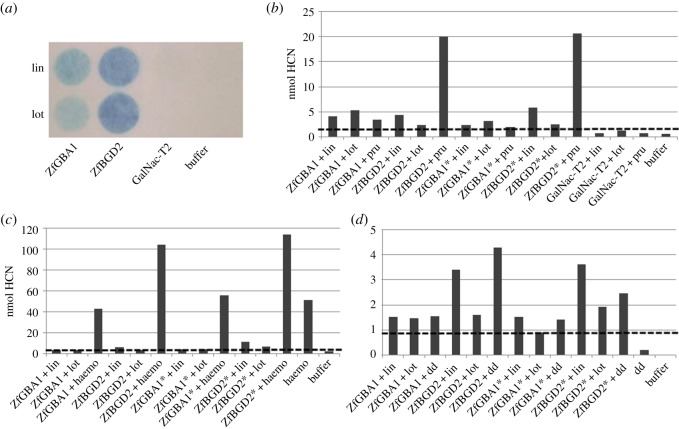
Enzyme activity of candidate β-glucosidases expressed in Sf9 cells with CNglcs as substrates. Cyanide emission from β-glucosidases was measured using Feigl-Anger paper (*a*) or colorimetric assays (*b*), spiked with boiled haemolymph (haemo) (*c*) or defence droplets (dd) (*d*). Only values above dashed lines are significant (based on controls). Linamarin (lin), lotaustralin (lot), prunasin (pru). Both boiled haemolymph and defence droplets contain lin and lot, but lack cyanogenic β-glucosidase activity. * enzymes expressed without putative native N-terminal signal peptide. The colorimetric assays were each repeated 4–6 times with different batches of expressed protein giving similar results, but separate experiments are not comparable due to different background emissions, so only one representative experiment of each type is shown.

To identify the enzyme responsible for hydrolysing CNglcs in *Z. filipendulae*, the β-glucosidases with the highest relative expression level according to the transcriptome sequencing, *ZfBGD2, ZfBGD3* and *ZfGBA1*, were chosen for heterologous expression in *Sf*9 insect cells*. ZfGBA1* and *ZfBGD2* were predicted to contain a native signal peptide and expressed both with and without (*) this peptide to ensure that the intrinsic N-terminal leader signal sequence in the expression vector would not be compromised. However, the recombinant proteins did not accumulate outside of the *Sf9* cells despite being fused to the leader signal sequence in the vector. Consequently, expressed enzymes were retained in *Sf9* cells, harvested by mild centrifugation, and the viability of cells was confirmed by FDA staining (electronic supplementary material, figure S2). Heterologously expressed enzymes of ZfBGD2 and ZfGBA1 were found to hydrolyse the generic substrate MUglc (ZfGBA1 ∼ 400, ZfBGD2 ∼ 3.000, GalNac-T2 ∼ 100 nmol released MU). ZfBGD2 and ZfGBA1 also hydrolysed linamarin and lotaustralin (figure 1*a*,*b*). Surprisingly, ZfBGD2 was found to hydrolyse the aromatic CNglc prunasin up to fivefold better than linamarin (figure 1*b*). When incubating the expressed candidates with boiled haemolymph (containing CNglcs but lacking β-glucosidase activity), only ZfBGD2 emitted twice the amount of HCN as compared to boiled haemolymph alone (figure 1*c*). Defence droplets containing CNglcs and a non-cyanogenic β-glucosidase [29], did not induce a significantly higher HCN emission upon addition of ZfBGD2 or ZfGBA1, indicating that a component from the larval haemolymph is needed to accelerate CNglc hydrolysis (figure 1*d*). To investigate this, haemolymph devoid of CNglcs, cyanogenic β-glucosidases or both was tested to see if it would increase HCN release from expressed proteins. No increase was observed (data not shown), so the component causing the effect from figure 1*c* must be lost during filtering of the haemolymph using a sample concentrator with 30 kDa cut-off and/or a desalting column.

### 3.2. ZfBGD2 transcript and enzyme are present in the haemocytes

**Figure 2.**
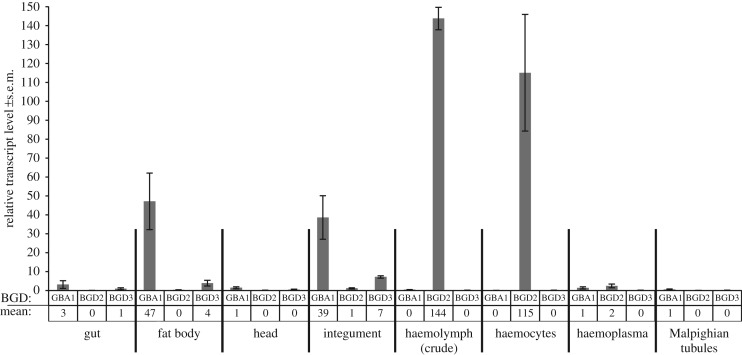
Relative gene expression levels of β-glucosidase candidates in different tissues of *Z. filipendulae* larvae measured by qRT-PCR. Normalized to reference gene *RpII140-RA*. Mean values of three to five biological replicates including standard error (s.e.m.) are given. For proteomics of haemocytes and haemoplasma see electronic supplementary material, file S3.

Gene expression of the three candidate genes in different larval tissues was analysed by qRT-PCR (figure 2). *ZfBGD2* expression was mainly detected in the haemocytes (mean 2^−ΔΔCt^-value of 115 ± 30.9 s.e.m.) while expression of the two candidates *ZfBGD3* and *ZfGBA1* were not detected here (mean 2^−ΔΔCt^-value of 0). A similar expression pattern was found in crude haemolymph. However, in the haemoplasma all three genes were either not or extremely lowly expressed. Instead, *ZfGBA1* was mainly expressed in fat body (mean 2^−ΔΔCt^-value of 47 ± 15.0 s.e.m.) and integument (mean 2^−ΔΔCt^-value of 39 ± 11.5 s.e.m.). Additionally, CNglc-containing integument (taken from the underside of the larvae, thus lacking cuticular cavities and haemolymph) released HCN after tissue homogenization in buffer. However, when integument was intact, no HCN release was detected, implying that ZfGBA1 is not in contact with linamarin and lotaustralin in this tissue. *ZfBGD3* generally showed low expression in the tissues analysed, having the highest expression in the integument with mean 2^−ΔΔCt^-values of 7 ± 0.7.

A total of 428 different proteins could be identified in the two fractions of haemolymph, of which 115 were only in the haemoplasma, 219 in the haemocytes, and 94 proteins were common in both fractions (electronic supplementary material, file S3). ZfBGD2 is detected only in haemocytes (coverage 25%) while ZfGBA1 is found in both fractions (coverage 64% in haemoplasma and 33% in haemocytes). ZfBGD3 is found only in haemoplasma (coverage 8%). Since both transcripts and proteins of ZfBGD2 are found in haemocytes, and not identified in the haemoplasma, it indicates that the protein is both produced and stored here. On the contrary, ZfGBA1 seems to be expressed and produced in the fat body and integument, and then at least a fraction of this enzyme is transported into the haemolymph.

### 3.3. Linamarin, lotaustralin and cyanide are present in the haemoplasma

**Figure 3.**
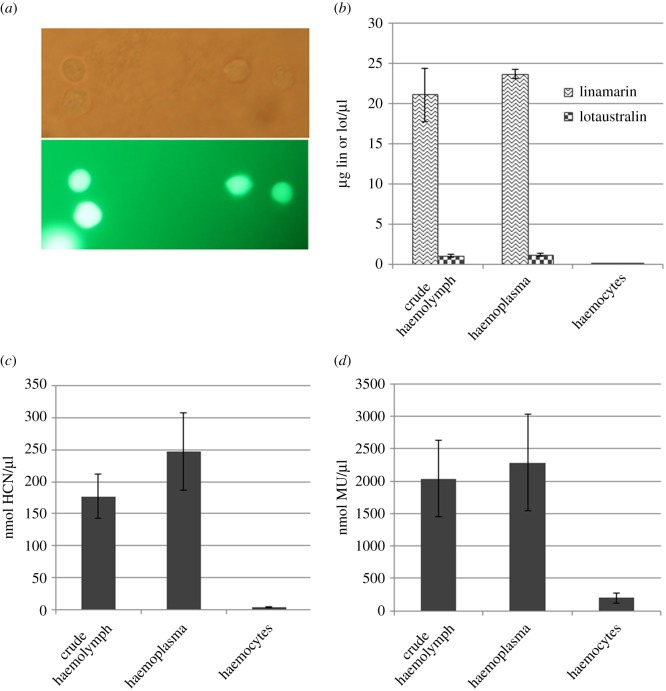
CNglcs and specific β-glucosidase activity present in crude haemolymph, haemoplasma and haemocytes. Haemocytes were intact after mild centrifugation, shown with FDA viability staining in the lower panel (*a*). Linamarin and lotaustralin (*b*), as well as specific (*c*) and generic (*d*) β-glucosidase activity was detected in crude haemolymph as well as haemoplasma. Neither CNglcs nor β-glucosidase activity was detected in haemocytes. Error bars show standard error (s.e.m).

To elucidate whether cyanogenic β-glucosidases and CNglcs are spatially separated within *Z. filipendulae* haemolymph, crude haemolymph, haemoplasma and haemocytes were analysed by LC-MS. Linamarin and lotaustralin were found in the haemoplasma in the same amount and ratio as in crude haemolymph (figure 3*b*), but not detected in the haemocytes. HCN emission was detected from haemoplasma and crude haemolymph at equally high levels, but not detected from haemocytes (figure 3*c*). Finally, generic β-glucosidase activity as monitored by the hydrolysis of MUglc was mainly present in haemoplasma and crude haemolymph as well, whereas only minor generic β-glucosidase activity was detected in haemocytes (figure 3*d*).

### 3.4. *ZfBGD2* evolved independently in insects compared to plants

**Figure 4.**
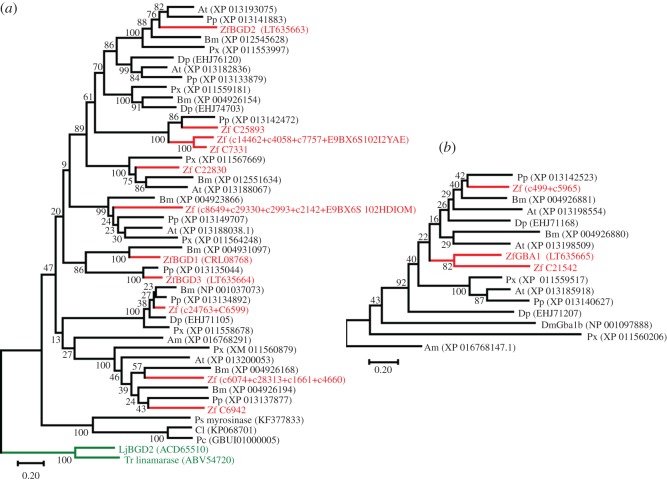
Models of the evolutionary history of insect GH 1 β-glucosidases (*a*) and GH 30 glucocerebrosidases (*b*). Analyses were carried out on protein sequences in MEGA7 using the maximum-likelihood method with a JTT model, and a discrete Gamma distribution. The branch lengths are measured in the number of substitutions per site, and 1000 bootstrap replicates were carried out. Am, *Apis mellifera*; At, *Amyelois transitella*; Bm, *Bombyx mori*; Cl, *Chrysomela lapponica*; Dm, *Drosophila melanogaster*; Dp, *Danaus plexippus*; LJ, *Lotus japonicus* (green); *Pc*, *Phaedon cochleariae*; Pp, *Papilio polytes*; Px, *Plutella xylostella*; Ps, *Phyllotreta striolata*; Tr, *Trifolium repens* (green); Zf, *Zygaena filipendulae* (red). (GenBank accession numbers shown).

Sequences belonging to GH family 1 and 30 were extracted from three *Z. filipendulae* transcriptomes, and each family was subjected to a phylogenetic analysis, with representative genes from lepidopteran species. *ZfBGD2* has close homologues from all butterflies and moths examined (figure 4), and clearly evolved independently in insects compared to plants, because the cyanogenic β-glucosidases from *L. japonicus* and *T. repens* plants do not cluster with the insect sequences. The few insect GH1 β-glucosidases involved in chemical defence which have been functionally characterized: a myrosinase (β-thioglucosidase) from *P. striolata* [10] and two β-glucosidases from Chrysomelina leaf beetles [11], formed their own cluster in the tree (45–50% identical to the other insect β-glucosidases) and were not closely related to any of the highly expressed β-glucosidases in *Z. filipendulae* (46–48% identical). *ZfBGD3* was the best hit when BLAST searching the *Z. filipendulae* transcriptomes with these enzymes as queries, although *ZfBGD2* was present in the top five hits (E-values 6 × 10^−89^, 2 × 10^−83^ and 4 × 10^−92^ respectively). *ZfGBA1* from *Z. filipendulae* has no close homologues from other lepidopterans, and is apparently non-existent in plants (http://www.cazy.org/GH30_eukaryota.html). It has been duplicated in *Z. filipendulae* recently, as seen by the closely related gene *Zf C21542*, which could indicate a new functionalization event.

To examine if the gene candidates had been submitted to selection, we calculated the nonsynonymous (d*N*) to synonymous (d*S*) substitution rate ratio (*ω* = d*N*/d*S*) on full-length sequences of β-glucosidases and glucocerebrosidases from *Z. filipendulae*. In our analyses, average *ω* values range from 0.00 to 0.53 (table 1). These values are all below 1, signifying purifying selection in both gene families. Model 6 (10 classes of *ω* values with 10% of the sequence fitted to each value, 2 gamma distributions) is the model with the best fit to the glucocerebrosidase sequences, and model 8 (10 classes of *ω* values with 10% of the sequence fitted to each value, *β* distribution, and one extra class of *ω* above 1) provides the best fit for the β-glucosidases. Both the models have many classes of *ω* values, which for the glucocerebrosidases are all below 1. This signifies that some segments of the glucocerebrosidase sequences are enduring more negative selection than others, but no sites with positive selection were detected. For the β-glucosidases 1.6% of the sequence was found to be likely under positive selection, although only one significant amino acid could be found in the empirical Bayes analysis (amino acid 36 from the alignment). The following amino acids with a non-significant probability were found as well: 17, 23, 40, 42, 574 and 575. No significant positive selection was found on any of the branches leading to the candidate genes (data not shown), so the positive selection found for the β-glucosidases seems not to be restricted to any specific clades in the phylogenetic tree.

**Table 1.** Nonsynonymous/synonymous substitution rate ratios (*ω*) in candidate genes from *Z. filipendulae.*

candidate genes	average *ω* (d*N/*d*S*)	best-fit model from PAML
β-glucosidases	0.13	Model 8: *p*: 10*0.10, 0.02
		*ω*: 0.00, 0.016, 0.03, 0.05, 0.07, 0.10, 0.13, 0.17, 0.23, 0.35, 1.94
glucocerebrosidases	0.18	Model 6: *p*: 10*0.10
		*ω*: 0.00, 0.03, 0.05, 0.08, 0.11, 0.15, 0.20, 0.25, 0.34, 0.53

### 3.5. Investigation of proposed positively selected residues

To analyse the importance of the proposed positively selected residues, a structural model of the ZfBGD2 protein sequence was produced by homology modelling. Two sequences were selected as templates and their structures used to create homology models. The spatial positioning of residues 23, 36, 40 and 42 hypothesized to be under positive selection was examined in the models. They all reside in the N-terminus of the protein, predicted to be a signal peptide, suggesting that the residues are unlikely to be important for protein function. This is supported by both templates retaining their function when lacking the approximately 20 residues of their N-terminus confirmed to be signal peptides. Unfortunately this truncation also made it impossible to model the proposed signal peptide region of ZfBGD2. Since ZfBGD2 is shorter than the alignment consensus sequence, residues 17, 574 and 575 are not present in ZfBGD2, and hence these residues could not be investigated further.

## 4. Discussion

Insect β-glucosidases are often studied for their ability to hydrolyse cellulose [45–48] or plant defence compounds [10,11,16,49–51], after isolation from digestive tissues such as salivary glands or gut [52,53]. Gut β-glucosidases from insects may also elicit indirect plant defences based on volatiles [54], or detoxify plant diterpene glycosides [55]. These studies underpin the general role of insect β-glucosidases in herbivore–plant interactions. Here, we elucidate another role of an insect β-glucosidase, i.e. involvement in cyanogenesis. Larvae of *Z. filipendulae* were used as model system to understand how and where in the insect body the CNglcs linamarin and lotaustralin are enzymatically hydrolysed to release HCN. The haemoplasma was identified as the part of the body with HCN release *per se*. The spatial separation of the cyanogenic β-glucosidase ZfBGD2 (present in haemocytes) and its cyanogenic substrates (present in haemoplasma) elucidated here, provides the basis for active cyanogenesis in the *Z. filipendulae* haemoplasma. We hypothesize that ZfBGD2 is released from haemocytes into haemoplasma, and at the same time rendered active perhaps by the loss of the putative inactivating signalling peptide, or by binding to another protein or other factor. Proteomic profiling did not detect ZfBGD2 in the haemoplasma (electronic supplementary material, file S3) but this does not exclude a low amount of the active enzyme here. Since HCN emission is restricted to the haemoplasma, direct cytotoxic effects to haemocytes are prevented. Moreover, *Zygaena* insects are generally resistant to HCN [56], mainly due to β-cyanoalanine synthase activity converting HCN and the amino acid cysteine into β-cyanoalanine [17,29]. Recently, β-cyanoalanine synthases involved in HCN detoxification were identified in other arthropods coping with HCN, such as the spider mite *Tetranychus urticae* [12] and the butterfly *Pieris rapae* [24]. The *Z. filipendulae* transcriptomes have also revealed a copy of this gene, although it has not been characterized yet.

Although there are many cyanide-releasing arthropods, including species from Chelicerata, Diplopoda, Chilopoda, Coleoptera, Heteroptera as well as Lepidoptera [17,19], ZfBGD2 is the first cyanogenic β-glucosidase characterized from an arthropod: it hydrolyses the two aliphatic CNglcs linamarin and lotaustralin resulting in HCN release, but also the aromatic non-physiological CNglc prunasin. Prunasin is not naturally present in the food plant of *Z. filipendulae* larvae, but it can be sequestered by them [57], and may be ingested from food plants by other species of Zygaenidae (Chiharu Koshio 2016, personal communication). Only few insect GH1 β-glucosidases involved in chemical defence have been functionally characterized: a myrosinase from the striped flea beetle *P. striolata* hydrolysing sequestered aliphatic glucosinolates [10], and a β-glucosidase from glands in juvenile Chrysomelina leaf beetles hydrolysing e.g. sequestered salicin or de novo produced 8-hydroxygeraniol-*β*-D-glucoside [11]. None of the examined β-glucosidases from *Z. filipendulae* were closely related to any of these functionally characterized β-glucosidases (figure 4).

Similar to *Z. filipendulae* larvae, cyanogenic plants store CNglcs and specific β-glucosidase spatially separate, such as in vacuole versus apoplast or chloroplast [3]. This ensures that toxic HCN is only released after tissue damage, when a cyanogenic β-glucosidase comes in contact with its substrates [9]. Thus, the HCN release *per se* from haemoplasma of *Z. filipendulae* is different from cyanogenic plants since no obvious tissue damage to larvae is necessary for HCN formation. This is consistent with the finding that *Z. filipendulae* larvae constantly release HCN [26,53]. Given that the total concentration of haemocytes may vary in response to biotic stress as shown for a polyphagous caterpillar [58], nutritional or mechanical stress in *Z. filipendulae* may similarly result in enhanced levels of ZfBGD2 released from haemocytes enabling a higher turnover of CNglcs. Further examples of compartmentalization between glucosylated defence compound and activating glucosidase in insects are found in the aphid specialists *Brevicoryne brassicae*, *Lipaphis erysimi*. They store an endogenous myrosinase in crystalline microbodies and sequester glucosinolates into their haemolymph from their Brassica host plant [6,59]. Disruption of this compartmentalization results in the release of biologically active isothiocyanates. *P. striolata* also produces its own myrosinase [10], but it is still unclear how these beetles avoid hydrolysis of stored glucosinolates [10]. Therefore, a similar spatial separation as in *Z. filipendulae* can be envisioned.

Since CNglcs in *Z. filipendulae* are derived from either sequestration or biosynthesis, ZfBGD2 likely has a key role in regulating their overall levels [60]. Accordingly, it was found in transcriptomes from *Z. filipendulae* larvae regardless of whether they were biosynthesizing or sequestering CNglcs. Interestingly, *ZfBGD2* is 1.4 times higher expressed in larvae that are sequestering compared to larvae that are biosynthesizing, as seen by comparing transcriptomes from them [31]. This is not statistically significant, but could indicate that the turnover of CNglcs is perhaps slightly higher in sequestering larvae due to the need to maintain the correct ratio of linamarin and lotaustralin regardless of the ratio in the ingested material [59].

Cuticular cavities harbouring defence droplets in *Z. filipendulae* larvae do not release HCN [29], although they contain even higher concentrations of CNglcs than crude haemolymph: approximately 25 µg µl^−1^ versus approximately 11 µg µl^−1^, respectively [60]. This is because the only β-glucosidase present in the defence droplets (ZfBGD1) cannot hydrolyse linamarin and lotaustralin [29]. However, in case of severe injury of a larva, CNglcs in the droplets will come in contact with exuding haemolymph and thus mix with the ZfBGD2 enzyme identified in this study. This interplay leads to increased levels of HCN release (57 versus 31 nmol HCN/h/µl from haemolymph alone [29]), and is supported by the finding here that significantly more HCN is released from haemolymph spiked with recombinant ZfBGD2 than from haemolymph alone (figure 1*c*).

When the glycosyl moiety of linamarin or lotaustralin is cleaved by ZfBGD2, the resulting α-hydroxynitrile is able to spontaneously dissociate into HCN and a ketone due to the pH of 6.3 in the haemolymph of *Z. filipendulae* [29]. Below pH 6 an α-hydroxynitrile lyase would be needed for HCN release. Since α-hydroxynitrile lyase activity has been found in crude haemolymph from *Z. trifolii* [61], the presence of an α-hydroxynitrile lyase could perhaps accelerate cyanogenesis in the haemolymph. Since more HCN is released when ZfBGD2 is combined with crude haemolymph (figure 1*c*), the enzyme might act in sequence with another protein or other component not present in *Sf*9 cells or media for higher turnover. Future studies will reveal whether it is indeed an α-hydroxynitrile lyase or other factors which are needed for faster HCN release in *Z. filipendulae*.

Although ZfBGD2 is clearly the primary cyanogenic β-glucosidase in *Z. filipendulae*, ZfGBA1 also has activity against linamarin and lotaustralin. This candidate is only expressed in integument and fat body in *Z. filipendulae* larvae, but the enzyme can be found in haemocytes and haemoplasma. Both integument and fat body contain CNglcs, but HCN was only released from the integument if it was homogenized, which would lead to mixing of ZfGBA1 with linamarin and lotaustralin. This indicates that ZfGBA1 is not present in the same subcompartment as the CNglcs in this tissue. Therefore, the primary function of this glucocerebrosidase is perhaps not cyanogenesis, but rather another yet unknown function, which is supported by negative purifying selection to maintain the gene. A study in *D. melanogaster* indicates that the glucocerebrosidase gene DmGba1b plays a role in the metabolism of protein aggregates in the insect brain [62]. A similar function could be envisioned for ZfGBA1 or its homologues in *Z. filipendulae*, although they are only 34–38% identical to DmGba1b (figure 4). The activity of ZfGBA1 against CNglcs found in this study is the first such activity assigned to any glucocerebrosidase, and gives an indication of the capabilities of this type of enzyme in insects.

Our phylogenetic analyses of ZfBGD2 show that the cyanogenic β-glucosidases evolved convergently in plants and insects. This is similar to the evolution of the biosynthetic pathway of CNglcs in *Z. filipendulae* and plants [7], and highlights the fact that there are not many ways to biosynthesize or hydrolyse CNglcs, and that enzymes from specific families have to be recruited for the pathways. Since the positive selection found in the β-glucosidases is restricted to the putative signal peptide in the N-terminal of the proteins, it can be assumed that the function of activating CNglcs is ancient, needing to be maintained, and thus under negative purifying selection. Biosynthesis of linamarin and lotaustralin has also been hypothesized to be an old trait, at least within Zygaenidae, and most likely also in the common ancestor of butterflies and moths [15,63]. Accordingly, bouts of positive selection present upon recruitment of genes for the pathway have long since been masked by a long period of purifying selection to maintain the sequence intact, once the optimal sequence had been obtained [30]. The hydrolysis of CNglcs by a specific β-glucosidase in *Z. filipendulae* probably evolved at the same early time point or even earlier than biosynthesis, since a capacity for turnover of CNglcs is necessary for utilization of these compounds in the insect.

## 5. Conclusion

Activity of a cyanogenic β-glucosidase was shown to play a pivotal role for cyanogenesis in a specialized Lepidopteran. Spatial separation of ZfBGD2 and its two cyanogenic substrates within the haemolymph (haemocytes versus haemoplasma) enables *Z. filipendulae* larvae to have a constant turnover of CNglcs and thus HCN formation. This compartmentalization of the CNglc/β-glucosidase system is similar to the situation found in cyanogenic plants [64]. ZfBGD2 is to our knowledge the first characterized arthropod β-glucosidase involved in CNglc catabolism and has evolved in convergence compared to cyanogenic plant β-glucosidases.

## Supplementary Material

Figure S1

## Supplementary Material

Figure S2

## Supplementary Material

Figure S3

## Supplementary Material

Table S1

## Supplementary Material

File S1

## Supplementary Material

Additional file S2 Alignments of BGDs and GBAs

## Supplementary Material

Additional file S3

## Data accessibility

Additional data can be found in electronic supplementary material, and accession numbers of sequences used in this study are shown in figure 4.
