# Supplementary figures and images for: Spatial separation of the cyanogenic β-glucosidase ZfBGD2 and cyanogenic glucosides in the haemolymph of Zygaena larvae facilitates cyanide release
Source: R Soc Open Sci. 2017 Jun 28;4(6):170262. doi: 10.1098/rsos.170262 (PMC5493921; doi:10.1098/rsos.170262)

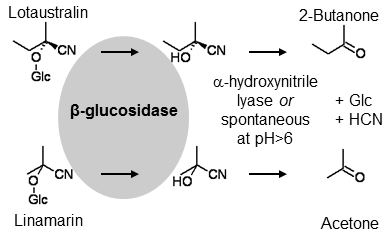

Supplement: Figure S1 [file rsos170262supp1.tif]

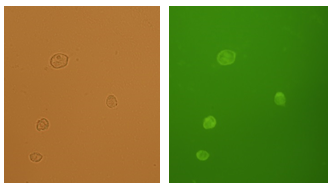

Supplement: Figure S2 [file rsos170262supp2.tif]

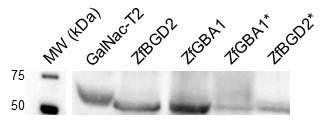

Supplement: Figure S3 [file rsos170262supp3.tif]
