# Supplementary material for: Spatial separation of the cyanogenic β-glucosidase ZfBGD2 and cyanogenic glucosides in the haemolymph of Zygaena larvae facilitates cyanide release
Source: R Soc Open Sci. 2017 Jun 28;4(6):170262. doi: 10.1098/rsos.170262 (PMC5493921; doi:10.1098/rsos.170262)
Supplement: Table S1 [file rsos170262supp4.docx]

Additional Table S1. Primer sequences used in this study. Restriction sites are underlined. 6xHIS-tag in italics.

| **Primer name** | **5‘-3’- sequence** |
| --- | --- |
| *For heterologous expression:* | |
| ZfGBA1orf*xma*I-F | TAAACCCGGGATGGCACATTTGGGGAGATCT |
| ZfGBA1orfHIS*not*I-R | AAAGCGGCCGCTCA*GTGATGATGATGATGATG*TGCCGCCGGTGCAGCCTTTGG |
| ZfBGD2orf*xma*I-F | TAAACCCGGGATGTGGTTCAGGTTGATCATC |
| ZfBGD2orfHIS*not*I-R | AAAGCGGCCGCTCA*GTGATGATGATGATGATG*GTATGTCCTGTCGATGGTCAT |
| ZfBGD3orf*xma*I-F | TAAACCCGGGATGAATAAGTTTTTATTGGCA |
| ZfBGD3orfHIS*not*I-R | AAAGCGGCCGCTCA*GTGATGATGATGATGATG*AAATTCGAGTGTGTAAGCTAA |
| *For qRT-PCR:* | |
| ZfGBA1qF | CAGCCCATGTTCTATGCCATG |
| ZfGBA1qR | CAAGAAAGCGACGTCACTGAC |
| ZfBGD2qF | CCACCGACTCATACCACAACTA |
| ZfBGD2qR | GGGAGGATCCTGGACCAAGA |
| ZfBGD3qF | AGGCGATTATCCCTCCGAGTTAA |
| ZfBGD3qR | CTGCCGTTCCCCGAATATAGTT |
