# Supplementary material for: Spatial separation of the cyanogenic β-glucosidase ZfBGD2 and cyanogenic glucosides in the haemolymph of Zygaena larvae facilitates cyanide release
Source: R Soc Open Sci. 2017 Jun 28;4(6):170262. doi: 10.1098/rsos.170262 (PMC5493921; doi:10.1098/rsos.170262)
Supplement: File S1 [file rsos170262supp5.docx]

Additional file S1

Western blots:

5μl 4x Laemmli buffer containing 1mM dithiothreitol was added to the samples and incubated at 95°C for 10 min to denature the proteins. Samples were applied to a Mini-PROTEAN^®^ 12% TGX stain-free gel (Bio-Rad) and run for 35 min at 200V in a 1X TGS buffer (Bio-Rad). Protein bands were visualized with the Chemidoc MP system (Bio-Rad). For western blotting, proteins were transferred to a Trans-Blot Turbo^®^ nitrocellulose membrane using the Trans-Blot Turbo system (Bio-Rad). The membrane was washed in PBST, blocked in 5% non-fat dry milk for 1 h, and washed three times in PBST for 5 min. The membrane was incubated with a conjugated (monoclonal anti-polyHistidine-peroxidase, A7058 Sigma) antibody diluted 1:2500 in PBS containing 1% bovine serum albumin for 1 h at RT. The antibody was removed and the membrane washed three times for 5 min in TBST. The blot was developed using Super Signal^®^ West Dura Extended Duration Substrate (Thermo Scientific) and visualized using the Chemidoc MP system.
